# Resistance mechanisms of bacterial biofilms on orthopedic implants and research progress on novel anti-biofilm coatings

**DOI:** 10.3389/fbioe.2026.1707232

**Published:** 2026-03-18

**Authors:** Xiaohang Liu, Pengcheng Ma, Xuan Liu, Shuai Liu, Xue Liu, Ruoyi Wang, Hongwei Gao

**Affiliations:** 1 The Second Qilu Hospital of Shandong University, Shandong University, Jinan, Shandong, China; 2 Shandong Public Health Clinical Center, Shandong University, Jinan, Shandong, China

**Keywords:** anti-biofilm coatings, antimicrobial drug-releasing coatings, bacterial biofilms, bioactive anti-biofilm strategies, implant-associated infections (IAIs), nanotechnology-based anti-biofilm strategies, quorum sensing (QS), surface physicochemical modification

## Abstract

Implant-associated infections (IAIs) have become a major challenge in clinical orthopedics due to the formation of bacterial biofilms and their complex resistance mechanisms. This review systematically summarizes the resistance mechanisms of bacterial biofilms on the surface of orthopedic implants and critically analyzes the research progress of novel anti-biofilm coatings. Novel antibiofilm coating strategies have shown a diversified development, which are mainly classified into: antimicrobial drug-releasing strategies, surface physicochemical modification strategies, nanotechnology-based antimicrobial strategies, and emerging bioactive strategies. Studies have shown that it is difficult to balance long-lasting antimicrobial activity and biocompatibility with a single strategy, and there is a need to develop multi-mechanism synergistic coatings (e.g., anti-adhesion, contact-killing, immune-modulatory) and to optimize the coating design by combining with artificial intelligence. Despite the potential of nanotechnology and bioactive strategies, their biosafety assessment, scale-up and long-term *in vivo* efficacy still need to be thoroughly investigated. This review provides an interdisciplinary perspective and theoretical basis for revealing the nature of biofilm resistance and developing efficient strategies for the prevention and treatment of IAIs.

## 1 Introduction

With the rapid development of modern medical technology and the intensification of aging, orthopedic implants (e.g., artificial joints, internal fixation plates, screws, etc.) have been increasingly used in the treatment of bone defects and functional recovery, significantly improving patients’ quality of life (Amin et al., 2020). However, IAIs have become one of the most serious complications after orthopedic surgeries. IAIs lead to the need for repeated surgeries, long-term use of antibiotics, prolonged hospital stays, reduced mobility, and even permanent disability (Tschon et al., 2019), which not only reduces patients’ quality of life, but also imposes a heavy economic burden on patients’ families and the social healthcare system (Hack et al., 2015). IAIs are characterized by the formation of bacterial biofilms on implants and surrounding tissues (Zimmerli et al., 2004). Biofilms consist of extracellular polymers (e.g., polysaccharides and proteins) mixed with bacteria (Onorato et al., 2024), which are highly resistant to the human immune system and traditional antibiotics. Biofilms endow bacteria with strong resistance, enabling them to evade antibiotic treatment and host immune clearance, leading to recurrent infections. In the face of increasingly severe bacterial resistance and biofilm infections, in-depth understanding of the resistance mechanisms of bacterial biofilms on the surface of orthopedic implants and, on this basis, research on novel and efficient anti-biofilm strategies—especially endowing implants with the ability to actively resist bacterial colonization and biofilm formation through surface modification or coating technology—has become a research hotspot and difficulty in the interdisciplinary fields of orthopedics, materials science, and microbiology (Akay and Yaghmur, 2024; Vallet-Regí et al., 2020). An ideal surface of orthopedic implants should not only have good biocompatibility and osseointegration ability but also excellent anti-infection performance to ensure the long-term stable function of the implants.

This review aims to systematically sort out the research progress on the resistance mechanisms of bacterial biofilms on orthopedic implants in recent years, and deeply explore the complex mechanisms related to drug resistance, such as the physicochemical properties of biofilms, barrier effects, special microenvironments, bacterial quorum sensing systems, specific resistance genes, and interactions with the host immune system. Meanwhile, we focus on the research of novel anti-biofilm coatings for orthopedic IAIs, and systematically summarizes the latest research results, mechanisms of action, advantages, and limitations of antimicrobial drug-releasing coatings, surface physicochemical modification strategies (e.g., anti-adhesion/antifouling coatings, contact bactericidal coatings), nanotechnology-based anti-biofilm strategies, and emerging bioactive strategies (e.g., bacteriophages, antimicrobial peptides, immunomodulation, etc.). Through the integration and analysis of existing literature, this review provides a theoretical basis and research ideas for revealing the fundamental principles of biofilm resistance, guiding rational clinical drug use, developing more effective strategies for the prevention and treatment of IAIs, and designing next-generation orthopedic implants with excellent biological functions and anti-infection performance.

## 2 Review

This manuscript is submitted as a Review article and provides an overview of orthopedic implant-associated infections, focusing on biofilm resistance mechanisms and anti-biofilm coating design. We summarize key resistance pathways and then discuss recent coating strategies and future directions.

## 3 Formation and resistance mechanisms of bacterial biofilms on orthopedic implants

### 3.1 Formation of bacterial biofilms on orthopedic implants

Bacterial biofilms are not simple aggregates of bacteria, but a highly structured and dynamically changing microecosystem (Dufour et al., 2010). Its core structure is the extracellular polymeric substances (EPS) matrix. EPS is mainly composed of polysaccharides, proteins, lipids, and extracellular DNA (eDNA), etc. EPS helps bacteria capture and accumulate nutrients from the environment and protects them from toxic metals and antimicrobial compounds. Meanwhile, EPS has significant diversity, and its component ratio varies with bacterial species, strains, and biofilm types. This diversity enables biofilms to adapt to different environments according to environmental signals and quorum sensing (Ramírez-Larrota and Eckhard, 2022).

**FIGURE 1:**
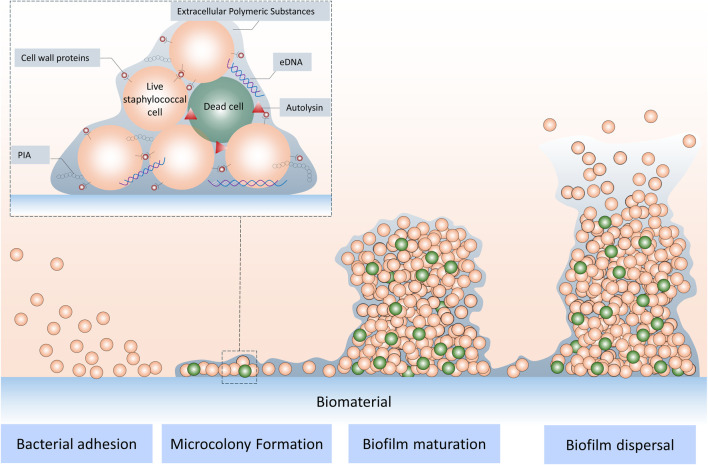
Stages of staphylococcal biofilm formation.

The formation of biofilms usually goes through several stages (Figure 1): (1) Bacterial adhesion: planktonic bacteria adhere to the implant surface; (2) Proliferation and microcolony formation: adhered bacteria begin to proliferate, forming small colonies; (3) Matrix secretion and maturation: bacteria secrete a large amount of extracellular polymers (EPS), forming a three-dimensional structure that wraps the bacteria, and the biofilm gradually matures; (4) Biofilm dispersal: some bacteria in the mature biofilm detach and become planktonic again, spreading to new sites to cause infections (Arciola et al., 2018). This process is affected by various factors such as bacterial species, surface properties of implant materials (e.g., roughness, chemical composition, hydrophilicity, etc.), and host environment (e.g., hematoma, tissue fluid composition).

### 3.2 Physical and chemical barrier effects

Zhou et al. (2015) considered biofilm as the first line of defense of bacteria against antimicrobial agents. The most direct mechanism of biofilm resistance comes from the physical barrier constituted by EPS. Dense EPS can significantly impede the penetration and diffusion of antibiotic molecules into the biofilm (Dufour et al., 2010; Arciola et al., 2018; Ansari et al., 2015). The penetration of macromolecular antibiotics (e.g., vancomycin) is particularly difficult, and even the concentration of small-molecular antibiotics in the biofilm is much lower than the effective bactericidal concentration.

In addition to physical barriers, some components in EPS may also chemically interact with antibiotics. For example, negatively charged polysaccharides or eDNA may bind to positively charged antibiotics (e.g., aminoglycosides), fixing them on the surface layer of the biofilm and preventing them from reaching the deep bacteria (Joshi et al., 2020). In addition, enzymes secreted by bacteria in biofilms (e.g., β-lactamase) are embedded in the EPS matrix, degrading or inactivating antibiotics before they reach bacterial cells.

These barrier effects indicate that effective anti-biofilm coatings for orthopedic implants should be designed to enhance penetration through the EPS matrix or locally disrupt its physicochemical integrity, rather than relying solely on increasing antibiotic dosage.

### 3.3 Biofilm microenvironment and changes in bacterial physiological states

Unlike fast-growing planktonic bacteria, most bacteria inside biofilms are in a slow-growing or even dormant state (Arciola et al., 2018; Mirzaei et al., 2024). This low metabolic activity makes them naturally insensitive to many antibiotics (e.g., β-lactams, quinolones) that require active bacterial proliferation to function. In addition, there is a small subset of cells in biofilms called “persister cells” (Arciola et al., 2018; Mirzaei et al., 2024). During antibiotic treatment, most bacteria are eliminated, but “persister cells” can tolerate extremely high concentrations of antibiotics and remain dormant neither growing nor dying. After the antibiotics are discontinued, the surviving persister cells participate in the reconstruction of biofilms, leading to chronic infections (Arciola et al., 2018; Mirzaei et al., 2024).

In addition to the altered physiological state, the biofilm environment may also promote the expression and spread of bacterial resistance genes. The efflux pump is a protein system that actively pumps antibiotics out of the bacterium, reducing intracellular drug concentrations. It is one of the important drug resistance mechanisms in bacteria (Gupta et al., 2017).

The presence of metabolically dormant and persister cells within biofilms highlights the need for coating strategies that target bacterial stress tolerance and microenvironmental conditions, rather than mechanisms dependent on active bacterial proliferation.

### 3.4 The role of quorum sensing in the regulation of bacterial drug resistance

Quorum sensing (QS) is a chemical communication mechanism among bacteria, whereby bacteria monitor the density of the population by producing and sensing their own secreted signal molecules (autoinducers) and coordinate the expression of a series of genes when a certain threshold is reached (Hu et al., 2024). The QS system plays a key role in a wide range of physiological activities of bacteria, including biofilm formation, virulence factor production, adhesion and transfer, and bacterial movement (Hu et al., 2024; Striednig and Hilbi, 2022).

**FIGURE 2:**
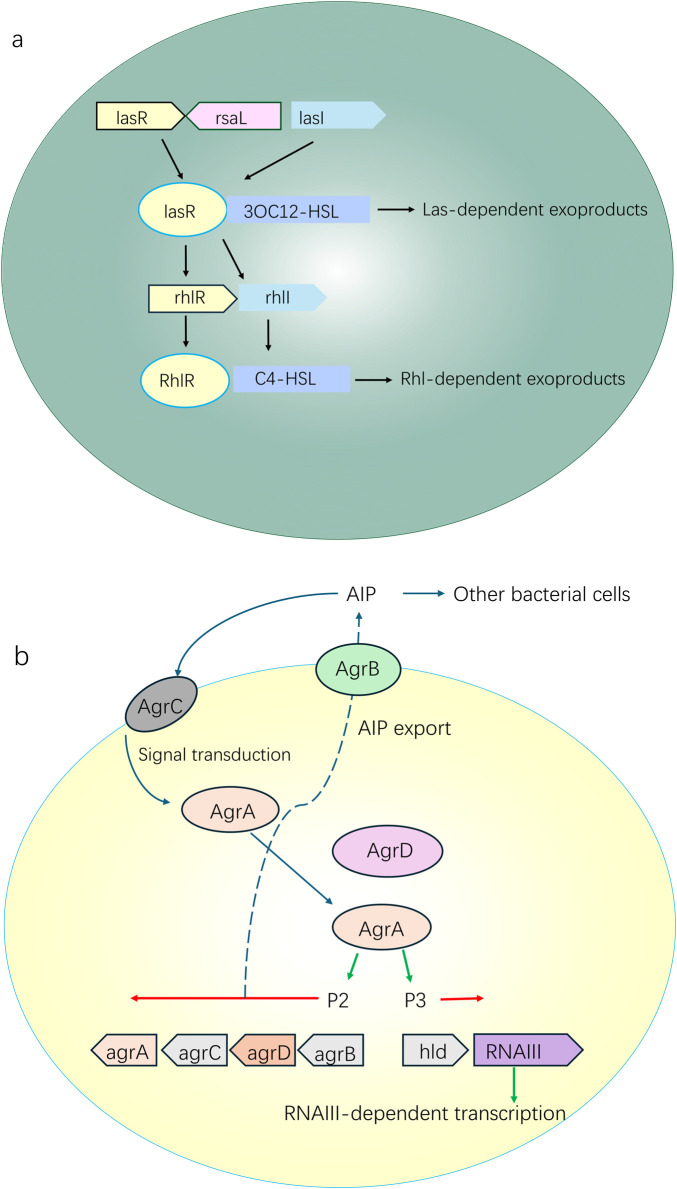
The QS system is closely related to the formation and maturation of biofilms: turn upregulates EPS matrix synthesis, promotes biofilm structure maturation, and expresses virulence factor-related genes: **(a)** the LasR-LasI and RhlR-RhlI systems of *Pseudomonas aeruginosa* induce the synthesis of extracellular proteases, alginate, etc., and promote the formation of biofilm matrix by regulating 3-oxo-C12-HSL and C4-HSL signals; **(b)** The agr system of *Staphylococcus aureus* regulates the expression of biofilm-related adhesion proteins (such as Fbe, ClfA) through AIP signals, and simultaneously inhibits matrix-degrading enzymes to maintain biofilm structure.

The QS system is closely related to the formation and maturation of biofilms. In the early stage of biofilm development, the concentration of QS signal molecules is low, and bacteria mainly adhere and initially aggregate. As the bacterial density increases, the concentration of QS signal molecules accumulates to a threshold, activating the QS system, which in turn upregulates EPS matrix synthesis, promotes biofilm structure maturation, and expresses virulence factor-related genes (Azimi et al., 2020). For example, the LasR-LasI and RhlR-RhlI systems of *Pseudomonas aeruginosa* induce the synthesis of extracellular proteases, alginate, etc., and promote the formation of biofilm matrix by regulating 3-oxo-C12-HSL and C4-HSL signals (Azimi et al., 2020). The agr system of *Staphylococcus aureus* regulates the expression of biofilm-related adhesion proteins (such as Fbe, ClfA) through AIP signals, and simultaneously inhibits matrix-degrading enzymes to maintain biofilm structure (Azimi et al., 2020) (Figure 2). In addition, QS induces phenotypic heterogeneity in bacterial populations, which drives biofilm structural differentiation, i.e., different subpopulations of cells perform different functions (e.g., matrix synthesis, diffusion, maintenance of drug resistance) and co-construct biofilms. For example, the Lqs system of *Legionella pneumophila* regulates the differentiation of intracellular survival subpopulations and motile subpopulations through LAI-1 signals; motile subpopulations promote the diffusion of biofilm edges, while persistent subpopulations maintain the core structure of biofilms (Azimi et al., 2020).

More importantly, the QS system can mediate the regulation of bacterial resistance (Azimi et al., 2020). On the one hand, the QS system directly regulates the expression of resistance genes: QS signals can activate efflux pump genes or modify antibiotic targets, reducing bacterial sensitivity to drugs. For example, LasR of *P. aeruginosa* can inhibit the expression of the β-lactam antibiotic target PBP3 and upregulate the MexAB-OprM efflux pump, enhancing drug resistance. QS can also induce the production of persister cells mentioned above. For example, *Legionella* regulates the proportion of persister cells in biofilms through the Lqs system, resulting in chronic infections (Striednig and Hilbi, 2022).

On the other hand, the QS system promotes metabolic cooperation among bacteria in biofilms (e.g., nutrient sharing) and transfers resistance genes through type secretion systems (T3SS, T6SS) or plasmids. For example, the QS system of *Vibrio cholerae* regulates the expression of T6SS, killing sensitive bacteria to release DNA and promoting horizontal transfer of resistance genes (Striednig and Hilbi, 2022).

Compared with resistance mechanisms that simply rely on physical barriers or changes in bacterial physiological states, the QS system provides a more active and coordinated regulatory network. Therefore, targeting the QS system in antimicrobial therapy may be more promising than simply destroying biofilm structures. However, different bacteria have diverse QS systems, and there are complex cross-interactions within the QS network and with other regulatory pathways, which makes the development of broad-spectrum and efficient QS inhibitors still face challenges (Hu et al., 2024; Azimi et al., 2020).

These findings suggest that incorporating quorum-sensing-interfering or signal-modulating components into implant coatings may effectively suppress biofilm maturation and resistance development without exerting strong selective pressure for classical antibiotic resistance.

### 3.5 Specific resistance genes and genetic factors

In addition to adaptive resistance conferred by biofilm structure and microenvironment, bacteria themselves carry resistance genes (Li and Webster, 2018). These resistance genes not only increase bacterial resistance to antibiotics, but also can be transmitted among bacterial populations through gene mutations and horizontal gene transfer (HGT), exacerbating the intractability and recurrence risk of infections (Santos et al., 2022; Sarkar et al., 2024).

Common resistance genes in bacteria include β-lactam resistance genes such as *mecA* and *blaZ*, as well as resistance genes to aminoglycosides and quinolones. They are prevalent in *S. aureus* and coagulase-negative staphylococci (CoNS), and are closely related to strains with strong biofilm-forming ability (Santos et al., 2022). Biofilm-related genes such as *icaADBC* and *icaR* promote biofilm formation, further enhancing bacterial defense against antibiotics and host immunity (Trobos et al., 2017). Other resistance-related genes such as *qacA* (antiseptic resistance gene), *ccrA/B* (recombinase gene), and *IS256* (transposase gene) are more common in clinically intractable cases (Santos et al., 2022; Harris et al., 2017).

In the biofilm environment, the high-density aggregation of bacteria provides favorable conditions for the horizontal transfer of resistance genes. Mobile genetic elements (MGEs) such as plasmids, transposons, integrons, and bacteriophages are key carriers mediating the transfer of bacterial resistance genes between different bacteria (even across species) (Montanaro et al., 2007).

In addition, scholars have recently discovered some new resistance mechanisms related to genetics. The latest studies have shown that the expression and regulation of bacterial ferritin genes not only affect iron homeostasis and antioxidant stress but also directly participate in the formation and regulation of antibiotic resistance. Oliveira et al. (2023) Oliveira et al. found that knocking out specific ferritin genes (such as *mycma_0076*) in mycobacteria leads to changes in cell wall structure, increased cell membrane permeability, significantly improved sensitivity to multiple antibiotics, and reduced resistance. Overexpression of ferritin genes can increase bacterial resistance to aminoglycoside antibiotics (e.g., amikacin and kanamycin), indicating that ferritin affects resistance by regulating iron homeostasis and stress responses (Khan et al., 2016).

Iron metabolism has also become a new target for anti-infection therapy. Ding et al. (2024) designed a nanomedicine based on layered double hydroxide (LDH), which systematically interferes with bacterial iron metabolism by releasing gallium ions (Ga^3+^) to replace iron ions (Fe^3+^) and iron chelators (DFP), thereby exerting antimicrobial effects and being able to act as a synergist for antibiotics (e.g., the iron-carrying cephalosporin Cefiderocol) to delay the development of resistance. This not only reveals the importance of iron metabolism in IAIs, but also provides a new strategy to combat bacteria (including drug-resistant bacteria) by interfering with key metabolic pathways.

The genetic basis of biofilm-associated resistance underscores the importance of multi-target or adjuvant-based coating designs that interfere with resistance gene expression, horizontal gene transfer, or key metabolic pathways, rather than single-mechanism antibacterial approaches.

### 3.6 Interaction between biofilms and host immune system

Biofilms on the surface of orthopedic implants not only resist antibiotics but also lead to persistent infections and even tissue damage through immune escape and regulation of host immune responses (Mirzaei et al., 2024; Seebach and Kubatzky, 2019; Mirzaei et al., 2019).

#### 3.6.1 Mechanisms of immune escape

Firstly, the EPS matrix of biofilms can act as a physical barrier, hindering the function of immune cells (e.g., neutrophils) and molecules (e.g., antibodies and complement) (Ramírez-Larrota and Eckhard, 2022). Secondly, the growth of biofilms can change the surface properties of microorganisms, reduce the recognition ability of immune cells, and alter cytokine responses, thereby inhibiting effective immune activation. In addition, some biofilm-forming bacteria secrete proteases, which participate in immune escape through various mechanisms such as degrading host immune molecules, interfering with immune signaling pathways, and damaging immune cell functions, ensuring the survival and proliferation of bacteria in the host (Bhattacharya et al., 2018). For example, *Helicobacter pylori* can secrete HtrA to destroy epithelial cell junctions and assist colonization (Ramírez-Larrota and Eckhard, 2022); *Streptococcus pyogenes* secretes SpeB, which can degrade various antibodies such as IgG, IgM, IgA, and IgD, with the hinge regions of IgG and IgD as main targets, inhibiting complement activation and antibody-mediated bacterial clearance (Ramírez-Larrota and Eckhard, 2022).

#### 3.6.2 Biofilm regulation of host immune responses

Biofilms can impede neutrophil migration and reduce the killing ability of innate immunity. Neutrophil extracellular traps (NETs) are meshwork composed of DNA, histones, and antimicrobial proteins released by neutrophils after stimulation by bacteria, fungi, etc., which can capture and kill pathogens (Mirzaei et al., 2019). Bhattacharya et al. (2018) revealed that MRSA biofilms release leukocidins such as PVL and HlgAB, which induced an abnormal release of NETs. The study found that leukocidins can deplete the phagocytic capacity of neutrophils and cause damage to surrounding tissues when inducing NETosis (the process of NETs formation), thereby helping bacteria in biofilms persist. In a porcine burn wound model, MRSA biofilms lacking PVL were more easily cleared by the host, indicating that targeting leukocidins is a promising direction for the treatment of chronic biofilm infections (Bhattacharya et al., 2018).

Biofilms can also actively induce an immunosuppressive microenvironment to evade immune surveillance and clearance. It has been found that at the site of chronic biofilm infections, there is often a concomitant recruitment and activation of immunosuppressive cells such as Myeloid-Derived Suppressor Cells (MDSCs), regulatory T cells (Tregs), and M2-type macrophages (Seebach and Kubatzky, 2019; Jiang et al., 2024; Wang et al., 2021). These cells can inhibit the activity of immune cells such as effector T cells, thereby weakening the host’s antimicrobial immune response (Akay and Yaghmur, 2024). Studies by Wang et al. (2021) and Jiang et al. (2024) have mentioned that the immunosuppressive microenvironment induced by biofilms is an important reason for the chronicity and recurrence of IAIs. The nanomaterial-based therapeutic strategies they proposed aim not only to directly kill bacteria and destroy biofilms but also to prevent infection recurrence by regulating immune responses (such as triggering macrophage-related immunity and reversing immunosuppression).

There is a complex interaction between biofilm infections and bone metabolism known as “osteoimmunology” (Seebach and Kubatzky, 2019). Chronic inflammatory responses caused by biofilm infections can stimulate osteoclast activation and bone resorption, and may inhibit osteoblast function and bone formation. This imbalance not only leads to osteolysis around implants and implant loosening but also may further exacerbate the infection process (Seebach and Kubatzky, 2019).

Compared with direct antibiotic resistance mechanisms, the interaction between biofilms and the immune system is a more complex dynamic process. Biofilms not only passively resist immune attacks but also actively shape the immune microenvironment to facilitate their own survival and persistence. This suggests that simple antimicrobial therapy may not be sufficient to eradicate IAIs, and combining immunomodulatory strategies to break the biofilm-induced immunosuppression and restore or enhance the effective antimicrobial immune response of the host may be an important direction for the treatment of chronic, refractory IAIs.

The complex interplay between biofilms and host immunity suggests that next-generation anti-biofilm coatings should not only focus on bacterial eradication but also actively modulate local immune responses to prevent immune evasion and infection recurrence.

## 4 Research progress on novel anti-biofilm coatings

There are various surface coatings for orthopedic implants, and different coatings can significantly improve the infection resistance, osseointegration ability, biocompatibility, and multifunctionality of implants. To facilitate comparison across major anti-biofilm coating strategies for orthopedic implants, we summarize their mechanisms, advantages, limitations, and translational considerations in Table 1. Reasonable selection and design of coatings can effectively reduce complications, prolong the service life of implants, and promote bone healing. Different anti-biofilm coatings and their research progress are described below.

**TABLE 1:** Comparative overview of anti-biofilm coating strategies for orthopedic implants.

Strategy category	Primary mechanism	Targeted biofilm stages	Key advantages	Main limitations	Evidence level/Translational status
Antimicrobial drug-releasing coatings	Local release of antibiotics or antibacterial adjuvants (Vallet-Regí et al., 2020; Kasapgil et al., 2024; Stewart and Costerton, 2001; Trampuz et al., 2003)	Early adhesion and initial biofilm formation	High local drug concentration; effective early infection prevention (Vallet-Regí et al., 2020; Kasapgil et al., 2024; Stewart and Costerton, 2001; Trampuz et al., 2003)	Limited duration; resistance risk; potential cytotoxicity (Vallet-Regí et al., 2020; Kasapgil et al., 2024; Stewart and Costerton, 2001; Trampuz et al., 2003)	Clinically applied (e.g., antibiotic-loaded bone cement (Tschon et al., 2019))
Anti-adhesion/antifouling surfaces	Inhibition of protein adsorption and bacterial attachment (Ghimire and Song, 2021; Ngo and Grunlan, 2017; Ishihara, 2022; Ishihara, 2024; Crago et al., 2024)	Initial bacterial attachment	Drug-free; low resistance risk; good biocompatibility (Ghimire and Song, 2021; Ngo and Grunlan, 2017; Ishihara, 2022; Ishihara, 2024; Crago et al., 2024)	Limited efficacy against strongly adhesive strains; durability concerns (Ghimire and Song, 2021; Ngo and Grunlan, 2017; Ishihara, 2022; Ishihara, 2024; Crago et al., 2024)	Preclinical/early translational (Ghimire and Song, 2021; Ngo and Grunlan, 2017; Ishihara, 2022; Ishihara, 2024; Crago et al., 2024)
Contact-killing surfaces	Surface-bound bactericidal agents disrupt bacterial membranes (Kasapgil et al., 2024; Celesti et al., 2022; Zhou et al., 2017)	Surface-attached bacteria	Long-lasting local activity; reduced systemic toxicity (Kasapgil et al., 2024; Celesti et al., 2022; Zhou et al., 2017)	Requires direct contact; activity decreases with surface fouling (Kasapgil et al., 2024; Celesti et al., 2022; Zhou et al., 2017)	Preclinical (Celesti et al., 2022; Zhou et al., 2017)
Nanotechnology-based strategies	ROS generation, catalytic reactions, or targeted delivery (Karnwal et al., 2023; Piruşcă et al., 2024)	Mature biofilms and dormant bacteria	Multifunctional; effective against drug-resistant bacteria (Karnwal et al., 2023; Piruşcă et al., 2024)	Biosafety concerns; fabrication complexity (Vallet-Regí et al., 2020; Karnwal et al., 2023; Piruşcă et al., 2024)	Preclinical (Karnwal et al., 2023; Piruşcă et al., 2024)
Physical therapy-based approaches (PTT/PDT/MHT/CDT)	Light-, magnetic-, or catalyst-triggered bactericidal effects (Jiang et al., 2024; Wang et al., 2021; Luo et al., 2024; Xu et al., 2024; Wang J. et al., 2024)	Established biofilms	On-demand activation; non-antibiotic mechanisms (Jiang et al., 2024; Wang et al., 2021; Luo et al., 2024; Xu et al., 2024; Wang J. et al., 2024)	Limited tissue penetration; equipment dependence (Jiang et al., 2024; Wang et al., 2021; Luo et al., 2024; Xu et al., 2024; Wang J. et al., 2024)	Preclinical (Jiang et al., 2024; Wang et al., 2021; Luo et al., 2024; Xu et al., 2024; Wang J. et al., 2024)
Bioactive and immunomodulatory strategies	Immune activation or microbial communication interference (Seebach and Kubatzky, 2019; Jiang et al., 2024; Wang et al., 2021; Morris et al., 2019; Galdiero et al., 2019; Hemshekhar et al., 2018; Yu et al., 2007; Alalwani et al., 2010; Wan et al., 2014)	Chronic and recurrent infections	Addresses immune evasion; reduces recurrence risk (Seebach and Kubatzky, 2019; Jiang et al., 2024; Wang et al., 2021; Morris et al., 2019; Galdiero et al., 2019; Hemshekhar et al., 2018; Yu et al., 2007; Alalwani et al., 2010; Wan et al., 2014)	Immune regulation complexity; safety concerns (Seebach and Kubatzky, 2019; Jiang et al., 2024; Wang et al., 2021)	Early-stage research (Seebach and Kubatzky, 2019; Jiang et al., 2024; Wang et al., 2021; Morris et al., 2019; Galdiero et al., 2019; Hemshekhar et al., 2018; Yu et al., 2007; Alalwani et al., 2010; Wan et al., 2014)
Synergistic multifunctional coatings	Integration of multiple antibacterial mechanisms (Jiang et al., 2024; Wang et al., 2021; Luo et al., 2024)	Multiple biofilm stages	Enhanced efficacy; reduced resistance development (Jiang et al., 2024; Wang et al., 2021; Luo et al., 2024)	Increased design and manufacturing complexity (Jiang et al., 2024; Wang et al., 2021; Luo et al., 2024)	Emerging research focus (Jiang et al., 2024; Wang et al., 2021; Luo et al., 2024)

### 4.1 Antimicrobial drug-releasing strategies

Antimicrobial drug-releasing coatings consist of an inert scaffold encapsulating one or more antimicrobial agents, which kill bacteria and inhibit biofilm formation through controlled local release of drugs at the implantation site (Kasapgil et al., 2024). Such coatings are typically composed of amorphous polymers above the glass transition temperature (such as poly(D, L-lactide) (PDLLA) and polylactic acid (PLA)), hydrogels, and porous ceramics (Vallet-Regí et al., 2020; Kasapgil et al., 2024). The advantages of this coating are that it can release high concentrations of antimicrobial drugs in the early stage of implantation when the infection risk is the highest, effectively preventing early infections, and can reduce the toxic side effects and resistance risks caused by systemic medication (Stewart and Costerton, 2001; Trampuz et al., 2003). The most commonly used antimicrobial drugs in antimicrobial drug-releasing coatings are antibiotics, including gentamicin, vancomycin, rifampicin, linezolid, and amoxicillin (Kasapgil et al., 2024).

Antimicrobial drug-releasing coatings also face challenges. The first is that drug release kinetics are difficult to control accurately, and an ideal release profile should provide adequate bacterial inhibitory concentrations during the period of high risk of infection (early postoperative period), with a gradual decrease in the later period to avoid long term toxicity and selective pressure for drug resistance (Tschon et al., 2019). Secondly, limited drug loading and release times usually provide only short-term protection and have limited effect on late-stage infections that may occur months or even years after implantation (Akay and Yaghmur, 2024). Thirdly, long-term low-dose antibiotic release may instead induce bacterial resistance (Li and Webster, 2018). Fourthly, some antimicrobial drugs or carrier materials may have cytotoxicity, affecting the osseointegration process (Vallet-Regí et al., 2020).

Therefore, achieving controlled release and long-term stability is a major challenge for antimicrobial drug-releasing coatings, and researchers are exploring more intelligent drug release systems. For example, characteristics of the biofilm microenvironment (e.g., low pH, specific enzymes) are used as triggers to achieve on-demand drug release (Ding et al., 2024). Ding et al. (2024) designed a nanomedicine named DFP@Ga-LDH-Cefi, which are loaded with iron chelator DFP and gallium ions are responsively released in the acidic microenvironment of the infection site, interfering with bacterial iron metabolism, while the loaded siderophore antibiotic cefiderocol can target bacteria. Chen et al. (2024) achieved on-demand release of ampicillin when the oligonucleotide (Oligo) linker secreted by micrococcal nuclease (MN) is cleaved, exploring the possibility of replacing broad-spectrum antibiotics, optimizing drug coupling, and engineering oligopeptide linkers to more effectively protect prostheses from polymicrobial infections.

In addition to upgrading coating materials, selecting appropriate antimicrobial drugs is also crucial. Considering the resistance of common strains in orthopedic infections (e.g., MRSA), it is necessary to select drugs with high sensitivity and low resistance potential, or adopt combination therapy strategies (Pfang et al., 2019). A study by Hanssen et al. (2024) explored the dose and duration of suppressive antimicrobial therapy (SAT) in orthopedic implant infections, finding that low-dose SAT is as effective as standard-dose SAT, and discontinuing SAT is safe in some patients. This suggests that when designing drug-releasing coatings, we may consider more optimized dose and release strategies, and even achieve limited-time release under specific conditions.

In summary, antimicrobial drug-releasing coatings are a direct and effective anti-infection strategy, especially suitable for preventing early infections. Future research directions lie in developing novel drug-loading systems with better controlled release performance, longer duration, smarter (e.g., environment-responsive release), lower toxic and side effects, and less tendency to induce resistance, combined with osseointegration-related functions (Vallet-Regí et al., 2020).

### 4.2 Surface physicochemical modification strategies

Surface physicochemical modification aims to enable implants to resist bacterial adhesion or kill contacting bacteria by changing their surface physical or chemical properties, thereby preventing biofilm formation at the source. This strategy does not rely on limited antimicrobial drugs carried by coatings, can provide longer-term protection, and may reduce the risk of resistance development. It mainly includes two categories: anti-adhesion/antifouling coatings and contact-killing coatings (Kasapgil et al., 2024).

#### 4.2.1 Anti-adhesion/antifouling coatings

**FIGURE 3:**
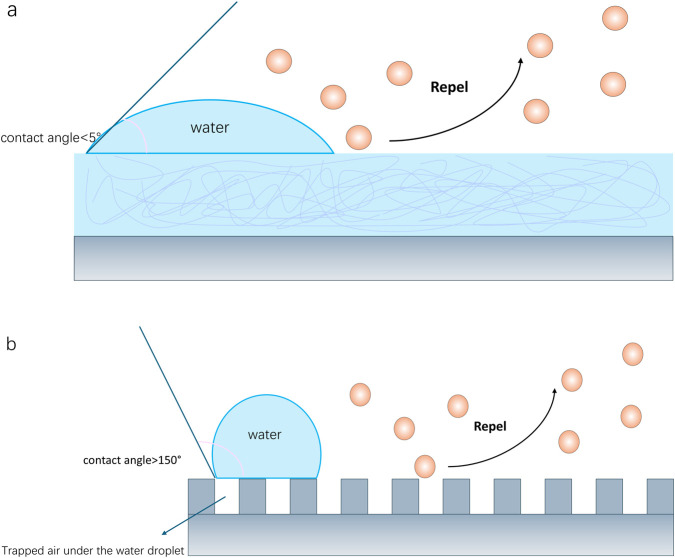
Anti-adhesion/antifouling coatings. **(a)** Superhydrophilic surface; **(b)** superhydrophobic surface.

Anti-adhesion and antifouling coatings have been extensively investigated as a non-drug-based strategy. The principle of such coatings is to construct an “inert” surface to minimize non-specific adsorption and adhesion of biomolecules such as proteins and bacteria, thereby inhibiting biofilm formation at the initial stage (Ghimire and Song, 2021; Ngo and Grunlan, 2017). The main ways to achieve anti-adhesion include superhydrophilic surface and superhydrophobic surface (Figure 3).

A superhydrophilic surface refers to constructing a strong hydration layer on the implant surface to minimize protein absorption and cell adhesion by reducing the contact area between the surface and water (Kasapgil et al., 2024). Polyethylene glycol (PEG) is a classic antifouling material, but it is easily oxidized and degraded *in vivo* and cannot be used for a long time (Ngo and Grunlan, 2017). In recent years, zwitterionic polymers, such as poly(phosphorylcholine) (MPC) and its derivatives, have attracted much attention due to their excellent biocompatibility, anti-protein adsorption, and anti-bacterial adhesion properties (Ishihara, 2022; Ishihara, 2024; Crago et al., 2024). Ishihara’s research introduced bionic material design based on zwitterionic polymers such as MPC and their application in surface modification of medical devices (including orthopedic implants). They can form a highly hydrated structure in the biological environment, effectively inhibiting biofouling and providing excellent lubricity (Ishihara, 2022; Ishihara, 2024). Crago et al. (2024) developed a method to stably graft zwitterions on polymer surfaces through plasma mediation, obtaining long-lasting superhydrophilicity, antithrombotic, and antifouling properties. Research on other hydrophilic polymers such as poly(ethylene glycol methyl acrylate) (POEGMA) and poly(N-vinylpyrrolidone) (PVP) has also made progress (Ngo and Grunlan, 2017).

Superhydrophobic surfaces achieve excellent water repellency and self-cleaning properties through the synergistic effect of micro-nano structures and low surface energy materials. It is characterized by a water contact angle greater than 150° and a droplet sliding angle less than 5° on a solid surface (Ghimire and Song, 2021; Souza et al., 2020; Erbil, 2020). Souza et al. (2020) prepared a superhydrophobic coating on titanium surfaces by one-step low-pressure plasma technology, improving the corrosion resistance of titanium, and significantly reducing bacterial and fungal adhesion and biofilm formation (by 8 times) *in vitro* experiments. In the oral *in situ* model, the coating changed the microbial composition of biofilms and significantly reduced the proportion of pathogenic bacteria related to peri-implantitis (by 7 times). This indicates that superhydrophobic surfaces can not only reduce biological load but also may create a more favorable microecological environment by selectively inhibiting harmful bacteria. However, the long-term stability of superhydrophobic surfaces, especially wear and contamination in complex *in vivo* environments, still needs further research (Ghimire and Song, 2021).

The main advantages of anti-adhesion/antifouling coatings are that they are not dependent on antimicrobial drugs, are less likely to induce resistance, and are usually well biocompatible. However, they cannot completely prevent the adhesion of all bacteria, especially for strains with strong adhesion ability, the effect may be limited. In addition, the long-term stability and wear resistance of coatings are also key issues to consider in clinical applications (Ghimire and Song, 2021; Crago et al., 2024).

#### 4.2.2 Contact-killing coatings

Cationic polymers are currently one of the most widely used contact-killing surfaces, using positively charged groups that electrostatically adsorb onto negatively charged bacterial cell membranes, thereby disrupting the integrity of the cell membrane and leading to bacterial death (Kasapgil et al., 2024; Celesti et al., 2022; Zhou et al., 2017). Most cationic polymer coatings contain chitin derivatives (such as chitosan), monomers with quaternary ammonium salts (QAC), or polyethyleneimine (Yi et al., 2022; Qiu et al., 2007). For example, medical-grade catheters coated with chitosan-based paint can significantly reduce biofilm formation and bacterial infections in mice (Hoque et al., 2016). Zhou et al. (2017) grafted cationic cross-linked coatings containing quaternary ammonium salts (AMPTMA) or quaternized polyethyleneimine (Q-PEI-MA) on the surface of silicone catheters through SI-ATRP (surface-initiated atom transfer radical polymerization) technology. This coating showed significant *in vivo* anti-biofilm and antimicrobial effects in a mouse catheter-related urinary tract infection model (reducing MRSA and VRE by 1.95 log and 1.26 log, respectively). Celesti et al. (2022) covalently grafted quaternary ammonium salts (QASs) with different alkyl chain lengths onto titanium surfaces, and found that long-chain QAS (such as Ti-AUTEAB) showed good anti-adhesion activity against *Escherichia coli* and *S. aureus*, especially in the early stage of contact (1.5 h), with a significant inhibitory effect on *E. coli*. These studies emphasize the importance of covalent fixation and molecular structure (e.g., alkyl chain length) for contact-killing effects.

Metals with antimicrobial activity (e.g., silver, copper, zinc) are fixed on the implant surface in the form of coatings or nanoparticles (Joshi et al., 2020; Hussein et al., 2024; Shkodenko et al., 2020; Luo et al., 2024). These metals can kill bacteria by releasing a small amount of ions or generating ROS through contact catalysis. Hussein et al. (2024) constructed a TiO_2_/Ag multilayer coating on the surface of Ti-13Nb-13Zr alloy through anodic oxidation and DC sputtering, aiming to use TiO_2_ to improve corrosion resistance and biocompatibility, and silver to provide antimicrobial performance. Luo et al. (2024) designed silicon-copper nanosheets (Si@Cu NSs), which utilize the photothermal properties of silicene nanosheets (Si NSs) and the Fenton-like catalytic activity of loaded copper nanoclusters (Cu NCs) to achieve photothermal-chemodynamic synergistic anti-infection therapy. They found that copper’s catalytic activity can be enhanced through p-d orbital hybridization. The antimicrobial effect of metal coatings is remarkable, but long-term release of metal ions may cause cytotoxicity, affect osseointegration, and even lead to heavy metal accumulation and environmental problems. Therefore, precise control of their dosage and release is required (Vallet-Regí et al., 2020; Shkodenko et al., 2020).

There are also other contact-killing mechanisms. Yu et al. (2024) developed a coating based on chiral metal-organic frameworks (MOFs). The coating is firmly anchored to the substrate through a metal-polyphenol network (MPN), and its MOF structure contains bactericidal Cu^2+^ ions and D-amino acid ligands with anti-biofilm activity. This chiral coating can not only kill bacteria through copper-catalyzed chemical kinetic reactions and inherent mechanical bactericidal activity, but also promote the disintegration of formed biofilms by releasing D-amino acid signals, achieving a synergistic antifouling mode of “first disassembly then killing”. The molecular mechanism of the broad-spectrum antibacterial and anti-biofilm effects was revealed by RNA sequencing analysis. This work demonstrates the potential of using chiral chemicals and MOF materials to design novel contact-killing coatings. Contact-killing coatings have stronger direct bactericidal ability compared to anti-adhesion coatings. Since antimicrobial components are fixed on the surface, they can theoretically provide more long-lasting antimicrobial effects and may reduce systemic toxicity caused by drug release (Kasapgil et al., 2024). However, the bactericidal effect depends on direct contact between bacteria and the surface, and their killing effect on bacteria at a distance or in thick-formed biofilms is limited. Due to the sensitivity of the coating surface to the accumulation of cells and cell debris, the accumulation of debris blocks the active surface, reduces its bactericidal effect, and provides anchor points for further bacterial attachment. Therefore, the antimicrobial activity of these coatings can only last for a short time, which is insufficient to completely eliminate infections, and further research is needed to enhance their long-term efficacy. It may be promising to combine this strategy with non-adhesive or cleaning mechanisms to reduce or remove accumulations on the surface (Kasapgil et al., 2024). In addition, long-term exposure to sublethal concentrations of fungicides (such as metal ions, cationic groups) may still select for resistant strains. The stability and wear resistance of coatings, as well as potential cytotoxicity, are also issues of concern (Celesti et al., 2022; Zhou et al., 2017).

Physicochemical modification strategies provide diversified anti-biofilm approaches. Anti-adhesion and contact-killing have their own advantages and disadvantages. The future trend may be to combine the two, such as designing surfaces with both anti-adhesion and contact-killing functions, or combining these modification strategies with other methods such as drug release and nanotechnology to obtain more comprehensive and long-lasting anti-biofilm effects (Akay and Yaghmur, 2024; Ghimire and Song, 2021).

### 4.3 Nanotechnology-based anti-biofilm strategies

The development of nanotechnology provides a new perspective and powerful tools for combating orthopedic implant-related infections and biofilms (Vallet-Regí et al., 2020; Karnwal et al., 2023). Due to the unique physicochemical properties of nanomaterials, such as small size effect, high specific surface area, and quantum size effect, they show great potential in antimicrobial, drug delivery, bioimaging, and tissue engineering (Wang D. Y. et al., 2024). In the field of anti-biofilm coatings, the application of nanotechnology is mainly reflected in the following aspects.

#### 4.3.1 Application of nano-antimicrobials

A variety of metal or metal oxide nanoparticles (NPs), such as silver (AgNPs), gold (AuNPs), copper oxide (CuO NPs), zinc oxide (ZnO NPs), titanium dioxide (TiO_2_ NPs), and iron oxide (Fe_3_O_4_ NPs), have been proven to have broad-spectrum antimicrobial activity and can effectively inhibit biofilm formation or destroy formed biofilms (Joshi et al., 2020; Karnwal et al., 2023; Piruşcă et al., 2024). Their antimicrobial mechanisms are diverse, including releasing metal ions to interfere with bacterial metabolism, generating a large amount of reactive oxygen species (ROS) to cause oxidative damage, and directly destroying cell membrane structures (Joshi et al., 2020; Karnwal et al., 2023). Joshi et al. (2020) summarized the interaction between gold and silver nanoparticles and bacterial biofilms, pointing out that these NPs can interact with biofilm components (polysaccharides, proteins, nucleic acids, lipids) through various forces such as electrostatic, hydrophobic, and hydrogen bonds, thereby destroying biofilm structures and inhibiting bacterial metabolism. Karnwal et al. (2023) systematically compared the anti-biofilm properties and mechanisms of various metal oxide NPs (CuO, Fe_3_O_4_, TiO_2_, ZnO, MgO, Al_2_O_3_). Piruşcă et al. (2024) prepared Fe_3_O_4_@SLS nanoparticles loaded with natural product Usnic acid (CAS: 125-46-2, UA) or antibiotic ceftriaxone (CEF) by co-precipitation method and made them into coatings by matrix-assisted pulsed laser evaporation (MAPLE) technology.

The advantages of nano-antimicrobials lie in their efficient and broad-spectrum bactericidal ability, as well as the potential to overcome traditional antibiotic resistance (Karnwal et al., 2023). However, their potential cytotoxicity, bioaccumulation, and possible induction of new nano-resistance still need in-depth research and evaluation (Karnwal et al., 2023; Almeida et al., 2024).

#### 4.3.2 Nano-drug delivery systems

Nano-drug delivery systems have attracted increasing attention for anti-biofilm applications, as they enable targeted, controlled, and stimulus-responsive delivery of antimicrobial agents within the complex biofilm microenvironment. Nanomaterials can serve as efficient drug carriers to target deliver antimicrobial drugs, gene fragments, or other bioactive molecules to infection sites or inside biofilms (Vallet-Regí et al., 2020). Nanocarriers, such as liposomes, polymer micelles, dendrimers, and inorganic nanoparticles such as mesoporous silica and LDH, can improve drug solubility and stability, prolong circulation time, and achieve controlled release or stimulus-responsive release (such as pH, enzyme, temperature, light, and magnetic field responsiveness), thereby improving efficacy and reducing toxic side effects (Vallet-Regí et al., 2020). Ding et al. (2024) used LDH nanosheets as carriers to co-load iron metabolism interfering agents (DFP, Ga^3+^) and antibiotics (cefiderocol), achieving responsive release and synergistic antimicrobial effects in the acidic microenvironment of infection sites.

#### 4.3.3 Surface topological structure engineering

This strategy affects bacterial adhesion behavior by constructing specific micro or nano-scale surface morphologies (Ghimire and Song, 2021). For example, nanopillar arrays that mimic the surface of a cicada wing can be “mechanical sterilization” (Hu et al., 2024). This method does not rely on chemical substances and is less susceptible to drug resistance. Hasan et al. used a chlorine-based reactive ion etching process to generate titanium nanopillars or black titanium, which can effectively kill *E. coli*, *P. aeruginosa*, *Mycobacterium* smegmatis, and *S. aureus*. However, its bactericidal efficiency may be affected by bacterial species, size, morphology, and nano-structure parameters, and large-scale preparation of uniform and stable nano-structures is still challenging (Hu et al., 2024).

#### 4.3.4 Nanomaterial-based physical therapy

Nanomaterial-based physical therapy has emerged as a non-antibiotic strategy that exploits the physical properties of nanomaterials (light, heat, and magnetism) to disrupt biofilms and eradicate bacteria. It mainly includes magnetothermal therapy (MHT), photothermal therapy (PTT), photodynamic therapy (PDT), and chemodynamic therapy (CDT).

Magnetothermal therapy (MHT) relies on the heat-generating capability of superparamagnetic nanomaterials under an alternating magnetic field to disrupt biofilm structures and eradicate bacteria. Superparamagnetic nanoparticles can efficiently generate heat under an alternating magnetic field (AMF), using local high temperature to kill bacteria and destroy biofilm structures (Wang et al., 2021). Wang et al. (2021) designed a magnetic synergistic therapy strategy based on CoFe_2_O_4_@MnFe_2_O_4_ magnetic nanoparticles (MNPs). Under AMF, MNPs generate enough heat to loosen the dense biofilm, and then MNP-SNOs coated with thermosensitive nitrosothiols penetrate into it, release NO, and efficiently kill bacteria under the synergy of magnetothermal effect. In addition, the nano-platform can also trigger macrophage-related immunity to prevent infection recurrence.

Photothermal therapy (PTT) utilizes photothermal nanomaterials to convert near-infrared light into localized heat for on-demand antibacterial action. Nanomaterials (e.g., gold nanorods, graphene, certain polymers, or dyes) can convert light energy into heat under near-infrared (NIR) light irradiation, achieving local high-temperature sterilization (Luo et al., 2024; Xu et al., 2024). Xu et al. (2024) successfully synthesized a “living” croconaine dye CR4EBiB, which has strong NIR absorption and high photothermal conversion efficiency. A variety of amphiphilic block copolymers were successfully synthesized by ATRP using CR-4EBiB as an initiator, and bifunctional antibacterial polymer coatings were constructed on PP by the volatilization film-forming method. The coating not only has antifouling properties but CR-4EBiB can also efficiently generate heat under NIR (808 nm) irradiation, effectively killing *E. coli*, *S. aureus*, and MRSA.

Photodynamic therapy (PDT) is based on photosensitizer-mediated reactive oxygen species generation under light irradiation to destroy bacterial cells and biofilm matrices. Photosensitizer (PS) nanoparticles transfer energy to surrounding oxygen molecules under excitation by light of a specific wavelength, generating reactive oxygen species (ROS) to kill bacteria and destroy biofilms (Jiang et al., 2024; Wang J. et al., 2024). Jiang et al. (2024) synthesized BSA nanoparticles (BMCV) loaded with manganese dioxide (MnO_2_), photosensitizer Ce6, and vancomycin. The nanoparticles can target and adhere to the biofilm infection area of *S. aureus*, catalyze H_2_O_2_ to produce oxygen in the hypoxic and acidic microenvironment of the biofilm, and enhance PDT effect. At the same time, ROS generated by light can degrade eDNA, destroy biofilm structure, lyse bacteria, and induce immunogenic cell death (ICD), activating adaptive immunity. Wang J. et al. (2024) loaded photosensitizers with aggregation-induced emission (AIE) properties into probiotic-derived bionic bacterium-like particles and fixed them on the substrate surface through plasma technology to form a coating. The coating generates ROS to kill bacteria under light irradiation and can regulate infection-related immune responses.

Chemodynamic therapy (CDT) uses nanomaterials (such as nanoenzymes containing Fe, Cu, Mn, etc.) to catalyze excess H_2_O_2_ in tumor or infection sites, and then undergo Fenton/Fenton-like reactions to generate highly toxic hydroxyl radicals (•OH) to kill pathogens. The Si@Cu NSs designed by Luo et al. (2024) utilize the Fenton-like catalytic activity of copper nanoclusters, and enhance their catalytic efficiency under physiological conditions through p-d orbital hybridization with silicene, achieving the synergy of CDT and PTT in anti-infection.

Nanotechnology-based strategies show great potential, especially in the development of multifunctional, intelligent, and synergistic anti-biofilm coatings. For example, integrating multiple functions such as antimicrobial, anti-adhesion, drug release, physical therapy (PTT/PDT/MHT/CDT), and immunomodulation into a single nano-platform is expected to achieve more efficient and long-lasting anti-infection effects and may overcome the resistance problem easily caused by single therapy (Jiang et al., 2024; Wang et al., 2021; Luo et al., 2024; Wang D. Y. et al., 2024). However, the biosafety of nanomaterials (including long-term toxicity, immunogenicity, *in vivo* metabolism, and clearance pathways) is a key issue that must be carefully evaluated before clinical translation (Karnwal et al., 2023; Almeida et al., 2024). In addition, the preparation process, stability, bonding strength with the substrate, and feasibility of large-scale production of nano-coatings are also very complex factors to consider.

### 4.4 Emerging bioactive strategies

While mature anti-biofilm coating strategies remain the clinical foundation for preventing orthopedic implant-associated infections, emerging bioactive and immunomodulatory approaches are increasingly explored as complementary solutions to address limitations of conventional designs. In addition to traditional chemical and physical methods, the use of biomolecules or biological agents to combat biofilm infections has attracted increasing attention. These strategies usually have high specificity, good biocompatibility, and may provide new mechanisms of action.

#### 4.4.1 Phage therapy

Phage-based strategies have re-emerged as a promising bioactive approach for implant-associated biofilm control, driven by advances in phage engineering and a renewed interest in precision antibacterial therapies. Bacteriophages are viruses that specifically infect and lyse bacteria. Using bacteriophages or their lytic enzymes to clear biofilm infections is an ancient yet revitalized strategy (Morris et al., 2019; Kaur et al., 2016). The advantages of bacteriophages lie in their high host specificity (only killing target bacteria without affecting normal flora), ability to self-replicate (amplify in the presence of host bacteria), and ability to evolve to overcome bacterial resistance. In addition, some bacteriophages can produce enzymes that degrade the EPS matrix, helping to penetrate biofilms (Morris et al., 2019).

Morris et al. (2019) evaluated the anti-biofilm activity of a phage mixture (StaPhage cocktail) against *S. aureus*. The phage mixture can effectively inhibit the growth of planktonic *S. aureus* and significantly reduce the number of viable bacteria in the formed biofilms on titanium scaffolds (by approximately 0.6 log10 CFU), while high concentrations of antibiotics have no effect on this biofilm. This indicates that bacteriophages have potential in treating staphylococcal biofilms on implants. However, bacteriophage therapy also faces challenges, such as possible development of phage resistance, standardization of phage preparation and purification, *in vivo* delivery efficiency, and potential immunogenicity (Morris et al., 2019).

#### 4.4.2 Immunomodulatory strategies

Immunomodulatory approaches have gained increasing attention in recent years, as accumulating evidence indicates that host immune dysregulation plays a central role in the persistence and recurrence of implant-associated biofilm infections. As mentioned earlier, biofilms can cause persistent infections and even tissue damage through immune escape and regulation of host immune responses (Seebach and Kubatzky, 2019; Jiang et al., 2024; Wang et al., 2021). Therefore, developing coatings or therapies that can regulate local immune responses, reverse immunosuppression, and enhance the host’s effective antimicrobial immunity has become a new research direction (Morris et al., 2019).

Host defense peptides (HDPs) are short, positively charged amphiphilic peptides naturally produced by the host’s innate defense system. They initially attracted attention due to their broad-spectrum antimicrobial activity, but recent studies have found that they also have multiple functions such as anti-biofilm, antiviral, immunomodulatory, and anti-inflammatory (Haney et al., 2019; Schneider et al., 2021; Haney et al., 2021). In the field of immune adjuvants, HDPs can act as modulators of the immune system, attracting immune cells, inducing cytokine secretion, and enhancing the effect of vaccines or other immunotherapies (Pachón et al., 2017; Fang et al., 2023).

The combination or synergistic application of metal particles (such as silver, zinc) and HDPs can enhance antimicrobial and immunomodulatory effects. Some studies have also explored the possibility of using metal nanoparticles as HDP delivery carriers or immune adjuvants. For example, covalently binding Cu^2+^ to synthetic HDPs (such as 1,018) and coating them on the surface of medical catheters can significantly inhibit the formation of *Staphylococcus epidermidis* biofilms, and reduce catheter-related infection rates by more than 60% in animal models (Haney et al., 2021). However, the related mechanisms and safety still need further research.

Seebach and Kubatzky (2019) (Jiang et al., 2024; Wang et al., 2021) discussed the potential of immunomodulation (e.g., targeting MDSCs, Tregs, or using immune checkpoint inhibitors) as a treatment for chronic implant-related bone infections. For example, using all-trans retinoic acid to reduce the number of MDSCs, or using INB03 to inhibit their proliferation and restore monocyte/neutrophil function.

Immunomodulatory strategies utilize or enhance the host’s own defense capabilities to combat infections, which may be more long-lasting and fundamental than simple bactericidal methods. However, the regulation of the immune system is extremely complex, and how to precisely and safely regulate local immune responses without triggering excessive inflammation or autoimmunity is the main challenge faced by this strategy.

#### 4.4.3 Antimicrobial peptides (AMPs)

Antimicrobial peptides have been widely studied as versatile bioactive agents with combined bactericidal, antibiofilm, and immunomodulatory properties, making them attractive candidates for implant surface functionalization. AMPs are small-molecule peptides produced by the natural immune system of organisms, with advantages such as broad-spectrum antimicrobial activity, rapid bactericidal effect, and low tendency to induce resistance (Galdiero et al., 2019). Many AMPs can not only kill planktonic bacteria but also effectively inhibit biofilm formation or destroy formed biofilms. Their mechanisms of action are diverse, usually involving interaction with bacterial cell membranes, leading to increased membrane permeability or membrane structure destruction, but they may also act on intracellular targets (Galdiero et al., 2019).

The bioactive peptide LL-37 is a product of human antimicrobial peptide hCAP 18 after cleavage. It promotes anti-infection responses by modifying the production of chemokines and pro-inflammatory factors and promoting the differentiation of monocytes into macrophages (Hemshekhar et al., 2018; Yu et al., 2007), and can also enhance the antimicrobial activity of macrophages and neutrophils (Alalwani et al., 2010; Wan et al., 2014). LL-37 can regulate the production of NETosis and reactive oxygen species in neutrophils, prevent biofilm formation by reducing bacterial adhesion and spread on implants, and play an important role in preventing staphylococcal infections. The immunomodulation of LL-37 can also promote the interaction between macrophages and osteoblasts, promoting osseointegration.

Galdiero et al. (2019) discussed the potential of AMPs as anti-biofilm drugs and pointed out the challenges faced in their application in the medical field, such as poor stability (easily degraded by proteases), low bioavailability, and high cost. To overcome these shortcomings, researchers are exploring combining AMPs with nanotechnology, such as loading them into nano-carriers or coupling them to nanoparticles to improve their stability, targeting, and efficacy (Galdiero et al., 2019).

#### 4.4.4 Strategies based on interference with microbial communication

Interfering with microbial communication pathways, particularly quorum sensing, has emerged as an alternative antibiofilm strategy that suppresses biofilm development without directly exerting bactericidal pressure. Since the QS system plays a key role in biofilm formation and resistance, developing coatings or drugs that can interfere with QS signaling pathways (QQ) can inhibit biofilm formation, reduce virulence, and may increase bacterial sensitivity to antibiotics. In addition, the chiral MOF coating developed by Yu et al. (2024) releases D-amino acids to promote biofilm disintegration, which also belongs to the strategy of interfering with bacterial population behavior.

#### 4.4.5 Natural biological interface materials

Biologically derived interface materials inspired by natural microbial communication and interaction mechanisms represent an emerging direction for regulating microbial behavior on implant surfaces. Zhou et al. (2024) proposed that outer membrane vesicles (OMVs)—signal components produced by bacteria themselves—can be used to construct implant coatings. They fixed OMVs derived from parental bacteria on the surface of various implants through a universal tannic acid bridging layer. This coating shows selective bacteriostatic ability: it can promote the proliferation of parental bacteria while inhibiting the growth of heterologous bacteria. Through high-throughput sequencing and bioinformatics analysis, they found that this selectivity stems from the ability of OMVs to upregulate antioxidant stress genes in heterologous bacteria (inhibiting their growth) and activate biofilm-related genes in parental bacteria (promoting their colonization). This study is the first to demonstrate the use of natural OMVs as biomaterials to achieve selective bacteriostasis through biological pathways, providing a new interface modification strategy based on natural bacterial communication mechanisms for regulating the microecological balance on implant surfaces. The advantages of this method lie in its potential high selectivity and biocompatibility, but the universality of its mechanism, long-term effect, and stability in complex *in vivo* environments still need further verification.

## 5 Summary and outlook

IAIs and their core pathogenic factor—bacterial biofilms—are major challenges in modern orthopedics. Biofilms endow bacteria with strong resistance, enabling them to evade antibiotic treatment and host immune clearance, leading to difficult-to-treat and recurrent infections, which seriously affect patients’ health and quality of life (Mirzaei et al., 2024; Li and Webster, 2018). The development of novel anti-biofilm coatings is an important strategy for the prevention and treatment of IAIs. This review systematically sorts out the complex mechanisms of bacterial biofilm resistance on orthopedic implants and reviews the research progress of anti-biofilm coatings. Moreover, to bridge technical parameters with real-world orthopedic needs, Table 2 maps representative clinical scenarios to corresponding coating design priorities and recommended strategies.

**TABLE 2:** Clinical scenario-oriented matching of anti-biofilm coating strategies in orthopedic implant-associated infections.

Clinical scenario	Major challenges	Design requirements	Recommended coating strategy
Early post-operative implantation	High bacterial contamination risk; acute inflammation	Rapid antibacterial activity; good biocompatibility	Antimicrobial drug-releasing coatings (Kasapgil et al., 2024; Stewart and Costerton, 2001; Trampuz et al., 2003)
Long-term implant retention	Late-onset infection; low-grade biofilms	Long-term stability; resistance avoidance	Anti-adhesion combined with contact-killing surfaces (Ghimire and Song, 2021; Ngo and Grunlan, 2017; Ishihara, 2022; Ishihara, 2024; Crago et al., 2024; Celesti et al., 2022; Zhou et al., 2017)
Multidrug-resistant infection	Antibiotic insensitivity; mature biofilms	Non-antibiotic mechanisms; deep biofilm penetration	Nanotechnology-based and physical therapy-assisted coatings (Jiang et al., 2024; Wang et al., 2021; Luo et al., 2024; Karnwal et al., 2023; Piruşcă et al., 2024; Wang J. et al., 2024)
Recurrent or chronic infection	Immune suppression; persistent biofilms	Immune modulation; sustained antimicrobial effects	Bioactive and immunomodulatory coatings (Seebach and Kubatzky, 2019; Jiang et al., 2024; Wang et al., 2021; Morris et al., 2019; Galdiero et al., 2019; Hemshekhar et al., 2018; Yu et al., 2007; Alalwani et al., 2010; Wan et al., 2014)
Revision surgery or high-risk patients	Compromised tissue microenvironment	Multifunctional and adaptive responses	Synergistic multi-mechanism coatings (Jiang et al., 2024; Wang et al., 2021; Luo et al., 2024)
Bone healing-critical applications	Infection-osseointegration conflict	Antibacterial activity without impairing osteogenesis	Dual-function anti-infective and osteoinductive coatings (Vallet-Regí et al., 2020)

Despite significant research progress, the development of ideal anti-biofilm coatings for orthopedic implants that can be widely used in clinical practice still faces many challenges. First, long-term effectiveness and stability are key. Coatings need to maintain their structural integrity and functional stability in complex *in vivo* physiological environments (mechanical stress, body fluid corrosion, enzymatic hydrolysis, etc.) to provide protection throughout the entire life cycle of the implant. Second, biocompatibility and osseointegration are basic requirements. Anti-biofilm coatings should not sacrifice host cell compatibility and osseointegration ability; ideal coatings should have both anti-infection and bone regeneration-promoting functions (Vallet-Regí et al., 2020). Third, broad-spectrum activity and resistance issues. Coatings should effectively combat various common pathogenic bacteria in orthopedics, including drug-resistant strains, and their mechanisms of action should not easily induce new resistance. Fourth, clinical translation and cost-effectiveness. Many new strategies perform well in the laboratory, but their preparation processes are complex, costs are high, and they are difficult to scale up for production and clinical translation. It is necessary to develop simpler, more stable, and more economical coating preparation technologies (Xu et al., 2024). Fifth, models and evaluation systems. Currently, there is a lack of standardized *in vitro* and *in vivo* evaluation models that can fully simulate complex *in vivo* environments (such as multi-species infections, host immune responses, blood flow shear force, etc.), which limits the accurate evaluation of the real effect of coatings (Morris et al., 2019; Galdiero et al., 2019).

Future research directions should focus on overcoming the above challenges, with emphasis on the following aspects.

1. Multifunctional synergistic strategies: Integrating different anti-biofilm mechanisms (such as anti-adhesion + contact sterilization, drug release + physical therapy, antimicrobial + immunomodulation) into the same coating system, using synergistic effects to improve anti-infection efficiency and durability, and reduce resistance risks (Jiang et al., 2024; Wang et al., 2021; Luo et al., 2024). At the same time, combining anti-infection functions with osseointegration-promoting functions (such as loading growth factors, constructing bionic structures) to achieve both “treatment” and “repair.”
2. Intelligent responsive systems: Developing intelligent coatings that can sense the infection microenvironment (such as pH, enzymes, specific biomarkers) and respond (such as on-demand drug release, activation of bactericidal functions) to achieve more precise, efficient, and safe treatment (Ding et al., 2024).
3. In-depth understanding of biofilm-host interactions: Further revealing the complex mechanisms of interactions between biofilms and the host immune system and bone metabolism system, especially the role of immunometabolism and osteoimmunology in IAIs, to provide a theoretical basis for developing novel anti-biofilm strategies based on immunomodulation and metabolic intervention.
4. Focus on multispecies biofilms: Real IAIs often involve mixed biofilms formed by multiple bacteria, whose structure, physiology, and resistance may be more complex than single-species biofilms (Mirzaei et al., 2024; Souza et al., 2020). Future research needs to pay more attention to the characteristics of multi-species biofilms and their prevention and treatment strategies.
5. Development of new bioactive materials and therapies: Continue to explore and optimize the application of biological agents such as bacteriophages, AMPs, enzymes, and OMVs, as well as new targets based on interfering with bacterial communication (QS, c-di-GMP, etc.) or metabolism (such as iron metabolism) (Ding et al., 2024).
6. Improvement of evaluation models and translational research: Establish *in vitro* and *in vivo* evaluation models that are closer to clinical reality, strengthen the connection between basic research and clinical applications, and promote the clinical translation of promising anti-biofilm coating technologies.

In conclusion, bacterial biofilm infections on orthopedic implants are a severe clinical challenge. In-depth understanding of the resistance mechanisms of biofilms is the basis for developing effective prevention and treatment strategies. In recent years, research on novel anti-biofilm coatings has made considerable progress, and technologies such as nanotechnology, drug-controlled release, surface engineering, bioactive molecules, and immunomodulation have shown great potential. Despite the remaining challenges, with the continuous interdisciplinary integration and innovation of materials science, microbiology, immunology, and clinical medicine, we have reason to believe that more safe and effective anti-infection orthopedic implants will be developed in the future, significantly reducing the incidence of IAIs and improving patient prognosis.
